# Supplementary material for: Role of reptiles and associated arthropods in the epidemiology of rickettsioses: A one health paradigm
Source: PLoS Negl Trop Dis. 2021 Feb 17;15(2):e0009090. doi: 10.1371/journal.pntd.0009090 (PMC7888606; doi:10.1371/journal.pntd.0009090)
Supplement: Attachment — Submitted filename: Rebuttal_PLOSNTD.docx [file pntd.0009090.s001.docx]

**Comments from reviewers:**

**Reviewer1**:

**Reviewer^’^s comment:** an interesting study which brings important new information on animals that are not been specifically addressed (reptiles and their ticks). need to better describe each group of animals or humans.

**Authors^’^ response:** Authors are thankful for the reviewer’s comments. In the revised version, an additional table with human patient data has been provided.

**Reviewer^’^s comment:** Needs to better describe the animals per species/number/ sex, adult and juveniles also give number of ticks and mites collected per animal
for humans: indicate from where were the 50 persons, sex age, profession.
need to indicate to which group the two persons with ticks on them belonged to.

**Authors^’^ response:** Additional information for animals and humans was added to the results section. In addition, information about humans was reported in a separate table.

**Reviewer^’^s comment:** we need a table that give more information on animals per species, age sex and location as well as number of ectoparasites collected on them. (can be expanded from Table 1). Same for humans (age, sex, location, profession) and who was infested with ticks (sex, age, location)

**Authors^’^ response:** Additional information for animals and humans was added to the results section. In addition, information about humans was reported in a separate table.

**Reviewer^’^s comment:** overall. discussion is acceptable. May be give some more information on previous report of Coxiella and Anaplasma in reptiles (Publications by Nieto et al., 2009 and Foley et al., 2016).

**Authors^’^ response:** More information about previous report of *Coxiella* and *Anaplasma* in reptiles have been reported in the discussion section and properly referred.

**Reviewer^’^s comment**: Line29: in a nature preserve park
Line 30: in an other Italian region.
Line 33: delete tails as it is confusing. You described it in your M $ M. Same for line 55
Line 38: antibodies anti--just antibodies to
Line 39: be more precise: Two ticks collected from 2 forestry workers
Line in the southern Italian Nature preserve park (delete herein)
Line 56: in questing ticks. delete from the environment
Line 57: althoug...lizards (plural)

Line 58: delete were
Line 68: the USA
Line 71: are known to
Line 73: confusing, as Coxiella is not a Rickettsiae
Line 78 delete the before Mediterranean
Line 81: site rather than place
Line 86: also known as TIBOLA.
Line 92: cite hepatitis before endocarditis, as hepatitis is usually during acute form and endocarditis in the chronic form.

Line 107: delete anti-
Line 108: in southern region of?? Italy?
Line 118: the collection rather than retrieval
Line 128: the study was..
Line 144: respectively not necessary

Lines 203, 204, 206: delete the before site and workers.

Line 219: Phases I and II

Replace by by "with" lines 233 and 235
Line 240: None of the four snakes...
Line 245: the only Rickettsial species identified using the ompA
Give the common names for the lizards: P. siculus: Italian wall lizard; P muralis: common wall lizard; Lacerta: Western green lizard; Elaphe: four line snake)

Line 255: thwo different Rickettsia species
Line 257: The two ticks..
Lines 261 and 264: which species?
Line 276: replace patients by subjects of whom four individuals
Line 287: suggestive of chronic Q fever. Reference 48 should be in the discussion.
Line 291: in a lizard population from Southern Italy (site 1)
Line 295: on them?? you mean humans?
Line 299: Present findings (delete herein presented
Line 301: Madeiran wall lizard (Teira dugesii)
Line 315: which also are
Line 316:delete "in turn"
Line 321 transmission in birds...
Line 343: from reptiles, ticks, and mites ...as well as ticks collected on two individuals...
Lines 347-348: This statement is out of context, as you indicate number of pet reptiles, but your study is on wild free-ranging reptiles in Italy. You should delete this sentence.

**Authors^’^ response:** All the corrections and comments above have been considered in the revised version of the text.

**Reviewer^’^s comment**: Line 93: what do you mean with Q fever being regionally restricted?? it is a very common disease everywhere, also more frequent in sheep/goat farming areas.

**Authors^’^ response:** The rationale for that statement is that earlier Q fever was considered as restricted but now it’s known to be widely distributed.

**Reviewer^’^s comment**: Line 151: need to better indicate the numbers. Was only one tick of 1 species collected from tow park rangers? it is my understanding that you had 2 ticks total collected on 2 persons? is it correct?

**Authors^’^ response:** The sentence has been modified.

**Reviewer^’^s comment**: Line 202: need to give, sex, age profession for your 50 individuals.

**Authors^’^ response:** An additional table has been provided in the revised version of the manuscript and discussed accordingly.

**Reviewer^’^s comment**: Results: numbers do not add with Table 1: I counted 168 animals for sites 1 and 2. You have 4 snakes Line 232 and 2 in Table (Line 251). It will be useful to know how many ticks were collected per snake/lizard or at list give range and mean).

**Authors^’^ response:** The numbers in the text and the table have been cross checked and are now consistent.

**Reviewer^’^s comment**: Lines 253-255: How many were positive for both genes?

**Authors^’^ response:** The samples positive for gltA gene was further screened with ompA gene thus the number of samples positive to ompA gene corresponds to the samples positive for both genes too.

**Reviewer^’^s comment**: For Anaplasma and Coxiella, you need to indicate that it is very uncommon in reptiles....data available from Nieto et al., 2009 and Foley et al., 2009

**Authors^’^ response:** Authors are thankful for reviewer’s valuable suggestion. The text has been changed accordingly and the two references have been added.

**Reviewer 3**

**Reviewer^’^s comment**: The integral view on this topic clearly merits publication, although the major drawback is the low sample number in all respective material. The data as well as the outcome does not justify that long list of authors and full paper. Methods are clear and state of the art. Results obtained are clear but low in number and are statistically on weak background. It is difficult to draw any conclusion by the low sample numbers investigated in this study.

**Authors^’^ response:** Authors are thankful to the reviewers for their comments. However, based on our own experience and the literature on the topic the number of reptiles, their ectoparasites and free-living ticks should be representative of the epidemiological context addressed in the current study. Also, the small number of human serum samples is because we used a convenience sampling approach. Even considering the small number of patients recruited, the fact that almost all the sampled patients were previously exposed to tick bites makes these samples very informative for the purpose of this study. Indeed, the prevalence of pathogens in these samples was very high, thus suggesting the human exposure to vector-borne pathogens of which reptiles could be reservoirs.

**Reviewer^’^s comment**: the role of N. autumnalis is rather vague in this respect, therefore I would omit this from the abstract.

**Authors^’^ response:** Authors agree with the reviewer in that the vector capacity of N. autumnalis needs to be further assessed. Nonetheless, given that lizards in the peri-urban site were solely infested by mites, larvae from this trombiculid mite could be involved in the enzootic cycle of these pathogens.

**Reviewer^’^s comments**:
P2L48 : Anaplasma phagocytophilum
P3L52: “One-Health”
P3L54:  Anaplasma phagocytophilum
P3L64: rephrase “how pivotal is…”
P3L69: Anaplasma phagocytophilum
P4L80: “… in Europe. Typical manifestations in human patients are …”
P4L90: “The Gamma- …”
P4l101: “…and is shed..”
P5L108: please clarify: “.. regions of “
P5L114: I miss Borrelia lusitaniae
P6L138: what time period
P7L159 bracket is missing.
P7L166: space befor 72°C
P8L188ff: genbank is registered trademark ®
P9L213. Rickettsiales and Legionellales

P16L376 “One-Health”

**Authors^’^ response:** All the corrections above have been considered in the revised version of the text.

**Reviewer^’^s comment**: P10L243: Maybe some statistics showing the relation eg. Kappa could help to understand the relations

**Authors^’^ response:**

Thanks for your comment and suggestion. These PCR assays target different genes and are well validated. It is acknowledged that not all gltA-positive samples will be ompA-positive. So, again thank you for your suggestion, but we believe that this comparative analysis is out of the scope of the present study and even not indicated .

**Reviewer^’^s comment**: P10l248 table 1: for me the table is confusing and I cannot understand the sense of it. It is not possible to distinguish the pathogens in respect to the different hosts (which line belongs to which host or vector…?)

**Authors^’^ response:** Thank you for your comment. Table 1 reports only the number and percentage of ectoparasites positive for *Rickettsia* spp. collected on reptile hosts. The description of the table has been changed in order to make it clearer. Host positivity was reported in the main text.

**Reviewer^’^s comment**: P14L330: N. autumnalis as potential vector is really vague and not proven. Furthermore the biology of this larval parasitism without any data on transstadial and transovarial transmission studies contradicts any possible transmission and therefor has to be handled with caution.

**Authors^’^ response:** Authors are thankful to the reviewers for their comments. Authors agree that further studies are needed to fully address the transstadial and transovarial transmission of rickettsiales in Nearctic Trombiculidae. We have added this to the manuscript.
